# Effectiveness of Virtual Reality Therapy on Static Postural Control and Dynamic Balance in Stroke Patients: Systematic Review, Meta-Analysis, and Meta-Regression of Randomized Controlled Trials

**DOI:** 10.3390/medicina62010090

**Published:** 2025-12-31

**Authors:** Ming-Yu Tian, Myoung-Ho Lee, Ju-Hak Kim, Myong-Kwon Kim

**Affiliations:** Department of Rehabilitation Sciences, Graduate School, Daegu University, Jillyang, Gyeongsan 712-714, Gyeongbuk, Republic of Korea; mingyutian5@gmail.com (M.-Y.T.); hotayaaa@gmail.com (M.-H.L.); rlawngkr2000@naver.com (J.-H.K.)

**Keywords:** virtual reality, postural control, stroke, rehabilitation, meta-analysis, randomized controlled trials

## Abstract

*Background and Objectives*: We aimed to evaluate the effectiveness of virtual reality (VR) therapy compared with conventional rehabilitation on static postural control and dynamic balance in patients with stroke. Design: A systematic review and meta-analysis of randomized controlled trials (RCTs) were conducted in accordance with the PRISMA 2020 guidelines. *Materials and Methods*: RCTs involving adults with stroke who received VR-based interventions, alone or combined with conventional therapy, were included. Outcomes were static postural control measured by center of pressure (COP) and dynamic balance assessed by the Berg Balance Scale (BBS) and Timed Up-and-Go Test (TUG). *Results*: Thirty-six RCTs (1118 participants) were analyzed. Pooled estimates favored VR-based interventions for several measures of static postural control, with standardized mean differences ranging from −0.59 to −0.38 (*p* < 0.05), whereas no clear difference was observed for COP sway speed under eyes-closed conditions (standardized mean difference (SMD) = –0.13, *p* = 0.43). For dynamic balance outcomes, pooled mean differences favored the VR group compared with conventional rehabilitation (BBS: mean difference (MD) = 3.29, 95% CI 2.76–3.83; TUG: MD = −3.43, 95% CI −4.03 to −2.82; both *p* < 0.0001). However, substantial heterogeneity and a high risk of bias were observed across several outcomes, which may affect the certainty of these findings. *Conclusions*: VR-based interventions may offer potential benefits for improving static postural control and dynamic balance in individuals with stroke, particularly when used as an adjunct to conventional rehabilitation. Nevertheless, given the substantial heterogeneity and risk of bias among included studies, the findings should be interpreted cautiously, and further well-designed, large-scale trials are required to confirm the magnitude and clinical relevance of these effects.

## 1. Introduction

Stroke is a non-communicable disease according to the latest Global Burden of Disease Survey 2021. It is the second leading cause of death and the third leading cause of death and disability [1]. Stroke is primarily classified into two main subtypes, hemorrhagic and ischemic, with ischemic stroke being the most common. Its pathogenesis involves atherosclerosis, cardiogenic embolism, and small vessel diseases [2]. Additionally, vascular rupture and the overall probability of stroke increase with age [3]. The incidence in young people has recently increased [4,5,6], possibly owing to increases in traditional modifiable risk factors, such as hypertension, diabetes, obesity, and hypercholesterolemia, as well as non-traditional factors such as migraine and smoking [4]. Considering the wide range of ages at onset, the ultimate rehabilitation goals also differ. Interventions to improve coordination, postural control, and balance should focus on older people with low demands to reduce falls and fall-related injuries [7]. Younger people with higher rehabilitation goals should improve their ability to perform daily activities to achieve the ultimate goals of rehabilitation, such as professional return and rebuilding the family’s role and responsibility [8].

However, the ultimate goal of rehabilitation requires that patients have good static posture control and dynamic balance ability. This is necessary to prevent further falls and pursue higher rehabilitation goals [9,10]. Neurodevelopment treatment (NDT), proprioceptive neuromuscular facilitation (PNF), task-oriented training, and walking and gait training in physical therapy are well-established routine interventions with clear evidence of improved balance and gait in stroke survivors: NDT therapy can improve balance and walking ability through trunk control training [11]; PNF therapy improves balance and gait speed by stimulating proprioceptive neuromuscular feedback loops, enhancing core stability and muscle strength, and improving motor coordination [12]; and task-oriented training significantly improves walking distance and speed by contextualizing tasks [13]. Treadmill training and weight-supported gait training are effective in improving walking speed and endurance in walking patients [14]. However, these approaches often rely on the professional experience of the therapist; the training context is relatively unitary, and patient engagement and motivation may be limited. In addition, traditional interventions face challenges in providing real-time, multisensory motor feedback and controlling labor and space costs during highly repetitive training.

Currently, virtual reality (VR) therapy is a new and rapidly developing treatment method. It mainly uses virtual reality technology alone to enable patients to interact with the virtual environment presented by the device to complete the intervention, which can provide visual, auditory, or tactile feedback to the user [15]. Early equipment is usually bulky and has low fitness, with a small operating space; however, recently, VR technology has developed rapidly. VR equipment has developed to be light, training content has diversified, the application cost is lower, and the population receiving VR therapy is also increasing [16]. Such a development trend provides diverse application scenarios in balance rehabilitation training for patients with stroke. In this era, understanding the specific therapeutic effects of VR in the rehabilitation of patients with stroke is urgently needed to promote it and provide valuable suggestions for the rehabilitation plans of more patients.

VR has a significant effect on balance rehabilitation in patients with chronic stroke and a positive effect on walking ability [17,18]. In addition, VR therapy has significantly influences the improvement of upper limb motor function in patients with stroke, especially immersive and specific-design VR therapy, which is mainly reflected in the improvement of motor function and hand flexibility [19,20]. These studies focused on the efficacy of upper- or lower-limb function; however, static postural control as the basis for ultimate rehabilitation goals has received little attention. Postural control is a complex skill based on dynamic sensorimotor processes, which involve active alignment of the trunk and head with gravity, support surfaces, visual environment, and internal references. This process relies on the integration of sensory information, including the somatosensory, vestibular, and visual systems. The results of this information integration can be monitored by measuring the swing amplitude, velocity, and other data of the center of pressure [21]. Postural sway in patients with acute stroke spontaneously increased, especially in the frontal plane [22,23]. Previous systematic reviews have investigated the evidence of homeostasis, and intervention duration, frequency, and functional recovery have been analyzed using meta-regression; however, small sample sizes, heterogeneity, and unsatisfactory statistical results have made the results inconclusive [24]. Further studies are required to evaluate the efficacy of VR therapy at different stages of stroke. These questions must be addressed if VR interventions are to be widely used to achieve better treatment outcomes.

This systematic review and analysis aimed to investigate the effectiveness of VR therapy in improving static posture maintenance and dynamic balance in stroke survivors. In addition, a meta-regression analysis was performed to explore the potential sources of heterogeneity across studies.

## 2. Materials and Methods

This study followed the Preferred Reporting Items for Systematic Reviews and Meta-Analyses (PRISMA) guidelines. The protocol has been published in the International Impact Research Registry of PROSPERO(CRD420251140599), accessible at https://www.crd.york.ac.uk/PROSPERO/view/CRD420251140599 (accessed on 28 December 2025).

### 2.1. Eligibility Criteria

The inclusion criteria were as follows: (1) randomized controlled trials (RCTs); (2) studies including participants who met the clinical diagnostic criteria for stroke or were diagnosed with stroke by magnetic resonance imaging or computed tomography and were medically stable and able to safely participate in exercise-based rehabilitation, without severe comorbidities or contraindications (e.g., severe cognitive impairment precluding task participation, unstable cardiac conditions such as decompensated heart failure, or other medical contraindications to exercise); (3) no restrictions on country, age, sex, or intervention duration; (4) virtual reality interventions used alone or in combination with other treatments, while the control group received conventional rehabilitation, including but not limited to physical therapy or traditional gait training; (5) outcome measures including center of pressure (COP) parameters in static standing (e.g., velocity, path length, sway area), the Berg Balance Scale (BBS), and the Timed Up and Go Test (TUG); and (6) only studies published in English were included.

The exclusion criteria were as follows: (1) single-group experimental design without a control group; (2) non-experimental studies, such as observational studies, case studies, qualitative studies, animal experiments, and duplicate publications, have reported the same results; (3) gray literature papers (abstracts and posters) that have not undergone peer review; (4) research lacking sufficient data for effect size analysis; and (5) the results of the report contain errors or inaccuracies in the tables or figures.

### 2.2. Search Strategy

The PubMed, EMBASE, Scopus, Web of Science, and Cochrane Library databases were searched, and the search was limited to between January 2015 and April 2025. The search strategy was developed jointly by members of the research team. Two investigators independently screened all study titles and abstracts after exporting the search results to all search tools and assessed the full text, with a second investigator consulted before deciding on controversial articles.

These search terms used were as follows: Stroke OR brain ischemia OR cerebral hemorrhage OR cerebrovascular disorders OR intracranial hemorrhages OR cerebrovascular accident OR post-stroke OR stroke rehabilitation AND virtual reality OR virtual reality training OR VR training OR virtual reality therapy OR real time feedback OR visual feedback OR immersion OR interactivity OR real-time rendering AND balance OR static balance OR dynamic balance OR center of pressure OR postural balance.

### 2.3. Study Selection and Data Extraction

Two researchers independently extracted data from the included RCTs, including the main author, country, year of publication, population characteristics (such as age and sex), study duration (minutes), name and model of VR intervention tools, description of VR intervention methods, description of control group intervention methods, measurement methods used, and main results.

In this review, VR intervention was defined as an intervention that employed computer generated or simulated environments allowing patients to interact with virtual scenarios in real time to perform balance or movement related tasks. Eligible VR interventions provided multisensory feedback, including visual, auditory, and/or haptic feedback, to facilitate motor learning and postural control. Both immersive and non-immersive VR systems were included, provided that the intervention involved active task engagement and real time feedback rather than passive observation. Interventions using simple video display without interactive components were not considered VR intervention.

### 2.4. Outcome Measures

We used four indicators—the COP path length (eyes open/eyes closed) and COP velocity (eyes open/eyes closed)—to comprehensively represent static postural control outcomes. The Berg Balance Scale (BBS) and Timed Up and Go (TUG) test were used to assess dynamic balance outcomes. Separate meta-analyses were performed for each of the six indicators.

### 2.5. Assessment of Risk of Bias and Quality of Evidence

The Cochrane risk of bias tool [25] was used to evaluate the methodological quality of the included studies. Each article was divided into low, high, and unclear risk of bias groups according to the following criteria: (1) bias arising from the randomization process (random sequence generation and allocation concealment); (2) bias due to deviations from intended interventions (blinding of participants and personnel); (3) bias in outcome measurement (blinding of outcome assessments); (4) bias due to missing (incomplete) outcome data; (5) bias in the selection of the reported results (selective reporting); and (6) other sources of bias (potential baseline imbalances, funding, and insufficient methodological reporting). A high-quality study must have five low-risk ratings and no remaining scores carrying a high error risk. A valid study must have at least four low-risk ratings, and no valid study must have high-risk ratings for any of the criteria.

The certainty of evidence was assessed using the Grading of Recommendations, Assessment, Development, and Evaluation (GRADE) framework, which classifies evidence into four levels: high, moderate, low, and very low [26]. Evidence may be downgraded according to five domains: risk of bias, inconsistency, indirectness, imprecision, and publication bias [27].

### 2.6. Statistical Analysis

A meta-analysis of the four measures of static postural control was performed using a random-effects model with standardized mean difference (SMD). A meta-analysis of the two indicators of dynamic balance was performed using a random effects model and mean difference (MD) [28,29]. SMD and MD were classified as large (>0.5), medium (0.3–0.5), small (0.1–0.2), or not significant (<0.1) between groups [30]. The Review Manager 5.3 statistical software (Cochrane Collaboration) was used to analyze the single effects of RCTs. The percentage of variation between studies due to statistical heterogeneity rather than chance was assessed using I^2^ statistics [28,29]. Percentage values close to zero indicate low levels of variation between studies [28,29]. The level of variation due to statistical heterogeneity was defined as follows: <40% was considered low, 30–60% moderate, 60–75% substantial, and >75% considerable heterogeneity [28,29]. Egger’s regression analysis was performed to assess the potential publication bias. Sensitivity analyses were performed for six measures. A trim-and-fill analysis was performed for the sensitivity analysis of indicators with more than 10 included studies, and model comparison was used for indicators with fewer than 10 included studies.

Meta-regression was used to explore the heterogeneity of the meta-analyses based on principles and linear regression. This study set a continuous covariate with four dichotomous covariates to account for sources of heterogeneity. The five covariates were VR intervention duration alone (minutes), stroke type (subacute stroke and chronic stroke), intervention type (VR intervention alone and VR intervention combined with other interventions), instrument used in VR intervention (Wii and non-Wii), and year of publication of the study (<2020 and ≥2020). Each model was fitted separately using the R software (R Foundation for Statistical Computing) via RStudio (version 2025.05.1+513; Posit Software, PBC), specifically the *metafor* package [31]. However, meta-regression analysis could not be used to explore the heterogeneity of the meta-analysis if fewer than 10 studies were included [28]. Therefore, a one-by-one removal was used to analyze the source of heterogeneity [29,32].

## 3. Results

### 3.1. Study Selection

**Figure 1:**
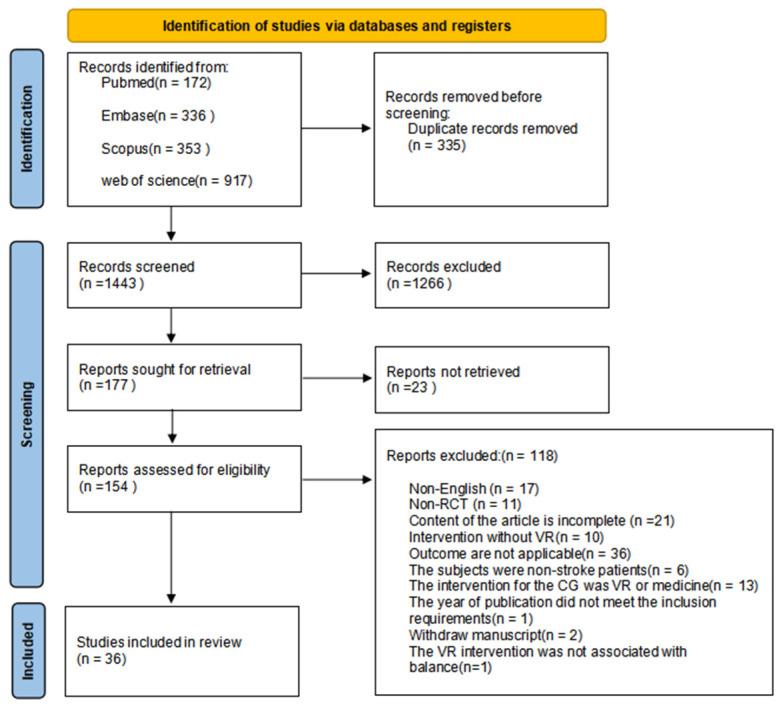
Flowchart of study selection.

In total, 1778 reports were retrieved and downloaded from the four platforms. After excluding duplicate articles that did not meet the inclusion criteria, 36 studies were included in the meta-analysis [33,34,35,36,37,38,39,40,41,42,43,44,45,46,47,48,49,50,51,52,53,54,55,56,57,58,59,60,61,62,63,64,65,66,67,68] (Figure 1).

The screening procedure is illustrated in the figure. During the screening process, the TUG data for Rooij 2021 were presented in the form of a geometric mean [69]. The method of converting the mean and SD values is complex and involves errors; therefore, it was excluded. In addition, although Dabrowska 2023 is an intervention using VR, and the results also measured BBS, its specific method of VR intervention is drawing training with VR, which is not related to balance; therefore, it was not included [70]. If the included studies were randomized to more than two groups using a VR intervention, the experimental group was preferentially included in a single VR intervention [64].

### 3.2. Characteristics of Study and Participant

A total of 36 RCT studies (1118 participants) were included in this review. Five studies on subacute stroke (<6 months), 20 studies on chronic stroke (>6 months), four studies on acute stroke (<3 months), and seven studies were unclear. Thirteen studies were on VR interventions alone, and 23 studies were on VR interventions combined with other interventions. Twenty-five studies used a single intervention in the control group, and 22 studies used a combination of two or more interventions in the control group. In 14 studies, the overall intervention time of the experimental group was longer than that of the control group, 1 study had a shorter overall intervention time of the experimental group than that of the control group, and the remaining 21 studies reported consistent intervention times of the experimental and control groups. The duration of the intervention was 2 weeks (1 study), 3 weeks (3 studies), 4 weeks (8 studies), 5 weeks (4 studies), 6 weeks (14 studies), and 8 weeks (6 studies). Fourteen types of tools were used in the VR intervention, among which the Nintendo Wii Fit was the most frequently used tool, and 13 studies used the Nintendo Wii Fit as a VR intervention tool. Another study did not clarify the model or name of the device used in the VR intervention [60]. For more detailed information, please refer to Supplementary Material.

#### 3.2.1. Description of VR Intervention

Balance training based on VR tools involves moving the weight to a specified position on a display, incorporating projections onto a treadmill for obstacle avoidance or weight shifting, or incorporating weight-loss systems, such as robots or protective ropes, to protect the patient during training. The main features are based on VR scenarios for center of gravity transfer, walking, or other balance training.

Games including ski simulation, catching balls, jumping over obstacles, and collecting items using virtual characters were used. The main features are more intuitive reward mechanisms, leveling mechanisms, or more vivid interactive scenes, requiring better endurance and attention of the object and providing sufficient fun.

Regarding virtual reality reflexology, the participants could observe and train the affected side by projecting the movement of the healthy side onto the affected side.

The tools used were mostly screen and interactive combinations, and immersion was not high. More immersive devices are typically characterized by their ability to create virtual characters, interact through glasses or larger screens, and provide more responsive real-time feedback. However, a unified advantage of VR intervention is that it is interesting, interactive, and reproducible.

#### 3.2.2. Description of the Control Intervention

Balance training included weight shifting, catching, walking in a straight line, crossing obstacles, and counterbalance training. It is characterized by weight shifting, confrontation, or walking training with manual assistance, robot assistance, or weight loss system assistance.

Physical therapy or traditional rehabilitation training included neurodevelopmental treatment, proprioceptive neuromuscular facilitation, Bobath, functional electrical stimulation, task-oriented training, and vestibular training. It is characterized by a mature theoretical basis and is widely used.

The exercise bike features bicycle training to increase lower limb muscle strength and coordination.

For sham VR reflexology, training was performed by projecting placebo images of lower limb movements onto the affected side.

### 3.3. Description of the Measurements

#### 3.3.1. Center of Pressure

Center-of-pressure (COP) outcomes were measured during quiet bipedal standing under both eyes-open and eyes-closed conditions. All COP-related data were converted into uniform units prior to inclusion in the meta-analysis.

COP path length was expressed in millimeters (mm) and COP velocity in millimeters per second (mm/s). COP path length was measured using the Prokin system [33,44], the Zebris force platform [57], and the Wii Balance Board [52,62]. COP velocity was assessed using the Huber 360 system [36], the Prokin system [44], and the Wii Balance Board [52,62].

Specific COP measurements were not included in the final analysis because they were too small. These data included the area of COP movement in the natural standing condition of both legs [44,57], the moving distance of the COP in the anterior–posterior (AP) or Medial–Lateral (ML) direction [54,57], and the distance and velocity of the COP while standing on both feet in a narrow range [62]. The speed of COP movement in the standing position with both feet forward and backward (tandem), or in the standing position with one leg was also recorded [34].

#### 3.3.2. Berg Balance Scale

The Berg Balance Scale contains 14 evaluation criteria that are mainly used to evaluate balance ability and fall risk (intraclass correlation coefficient [ICC] = 0.99) [70]. The lowest and highest scores are 0 and 56, respectively. A score of 0–20 points: severe balance disorder, extremely poor balance ability, high risk of falls, and limited ability to take care of oneself. A score of 21–40 indicates a moderate balance disorder in which the patient has impaired balance and limited mobility and requires assistance or monitoring. A score of 41–56 indicates mild or no balance disorder, good balance ability, strong self-care ability, and a low risk of falls.

#### 3.3.3. Timed Up-And-Go Test

The Timed Up-and-Go test is a commonly used tool to assess functional mobility and balance, and primarily measures the time in seconds (s) required for a participant to rise from a chair, walk 3 m, turn around, return to the chair, and sit down (ICC = 0.98) [71]. Lower scores indicate better functional mobility and greater balance and mobility. Results of ≤10 s indicated normal mobility, good balance, and low fall risk. A score of 11–20 indicates suitability for patients with frailty, older people, or persons with mild disabilities, within the normal range; however, falls may remain a risk. A time of 20 s indicates limited functional mobility, reduced balance, increased risk of falls, and may require assistance or rehabilitation intervention. A time of 30 s indicates difficult movement, severely limited function, and high risk of falling. The units of all TUG test results were collated before inclusion in the analysis.

### 3.4. Risk of Bias in Studies

**Figure 2:**
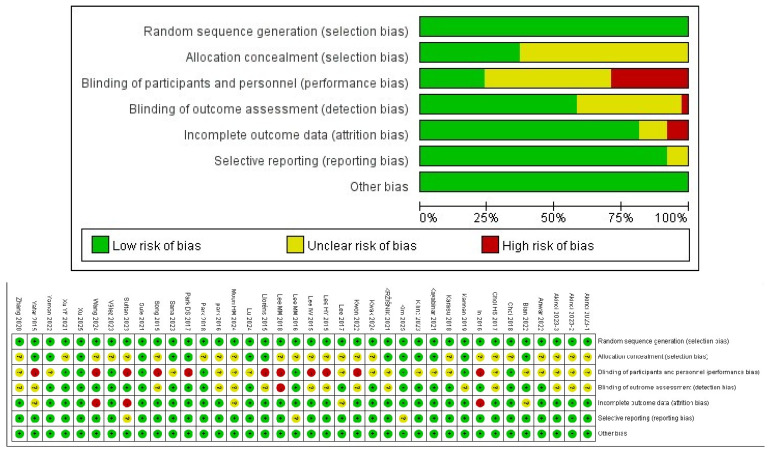
Risk of bias of the individual studies.Green circles indicate low risk of bias, yellow circles indicate some concerns, and red circles indicate high risk of bias; symbols within the circles reflect the corresponding judgments for each domain.

The Cochrane ROB 2 tool was used to assess the risk of bias in the included studies [25]. Seventeen studies were judged to have “some concerns,” mainly because of a lack of sufficient information on allocation concealment and blinding of outcome assessors. An additional 17 studies were judged to have a “high risk of bias” owing to deviations in intervention implementation or selective reporting of outcomes. Only two studies were judged to have a “low risk” in all domains (Figure 2).

1. Regarding the bias caused by the randomization process, 14 studies were considered to have a low risk, whereas 22 studies were judged as having certain concerns, mainly because of the unclear description of the allocation sequence concealment method.
2. Regarding bias due to deviation from the intended intervention, 8 studies were rated as having a low risk, whereas 17 studies were judged as having some concerns, and 11 were judged as having a high risk. This is attributable to the particularity of the VR intervention, which makes it difficult to achieve complete blinding of the participants and implementers.
3. Regarding the bias of outcome measures, 23 studies were rated as having a low risk, 13 studies were rated as having some concern, and no study was rated as having a high risk. This was mainly because of the lack of description of the assessors’ blinding.
4. Regarding bias due to missing outcome data, 28 studies were rated as having a low risk, 4 studies were rated as having some concerns, and 4 studies were rated as having a high risk. Some studies were considered high-risk because they had a dropout rate of >20% and did not select appropriate methods for analysis.
5. Regarding the bias of reporting results, 34 studies were considered to have a low risk, 2 studies were judged as having some concerns, and 0 was judged as having a high risk.
6. Among other biases, no potential baseline imbalances, funding sources, or insufficient methodological reporting in any of the studies were observed; therefore, all were judged as having a low risk.

### 3.5. Main Result: Static Posture Control

#### 3.5.1. Center of Pressure (COP) Sway Path Length with Eyes Open

**Figure 3:**
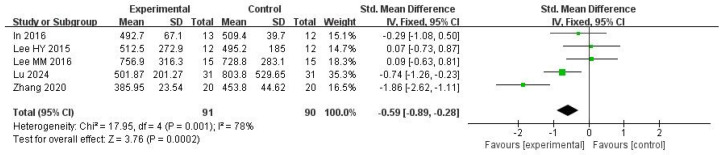
Standardized mean difference for the effectiveness of VR intervention compared with non-VR treatment for COP sway path length with eyes open. Green squares indicate individual study effects, with square size proportional to study weight, and horizontal lines represent 95% confidence intervals; the diamond represents the pooled effect estimate.

**Figure 4:**
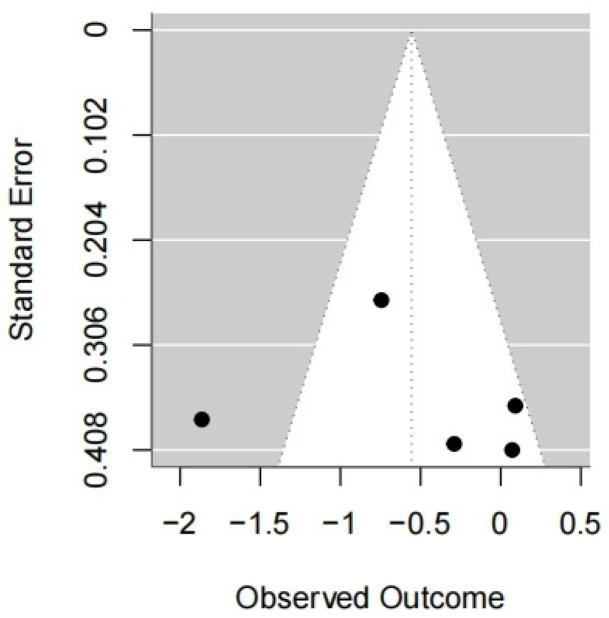
Forest plot of included studies in terms of COP sway path length in the eyes-open state.Each dot represents an individual study. The dotted vertical line indicates the pooled effect estimate, and the grey shaded area represents the 95% confidence region of the funnel plot.

Five studies were included in this meta-analysis. Under the random effects model, the VR intervention was associated with a reduction in COP sway path length under eyes-open conditions, with a pooled effect size of SMD = −0.59 (95% CI: −0.89 to −0.28; I^2^ = 78%; *p* = 0.0002) (Figure 3 and Figure 4).

Sensitivity analyses using alternative estimators of between-study variance showed a similar direction of effect; however, confidence intervals crossed the null value and statistical significance was not consistently observed (restricted maximum likelihood (REML): 95% CI −1.25 to 0.14, *p* = 0.12; DerSimonian–Laird (DL): 95% CI −1.23 to 0.11, *p* = 0.10)(Table 1).

Leave-one-out analysis indicated that the pooled effect size and its confidence interval remained consistent across all scenarios (ranging approximately from −1.52 to −0.73), and none of the results reached statistical significance (*p* > 0.05). Zhang (2020) [44] contributed substantially to the heterogeneity (I^2^ decreased from 80% to 40% when excluded). Thus, the high I^2^ could be largely driven by this study (Table 1).

Overall, the findings appear stable in direction but exhibit sensitivity in terms of heterogeneity, particularly with respect to the inclusion of Zhang (2020) [44].

#### 3.5.2. COP Sway Path Length with Eyes Closed

**Figure 5:**
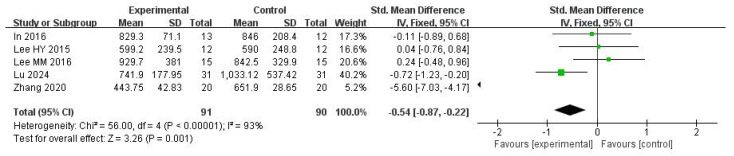
Standardized mean difference for the effectiveness of VR intervention compared with non-VR treatment for COP sway path length with eyes close. Green squares indicate individual study effects, with square size proportional to study weight, and horizontal lines represent 95% confidence intervals; the diamond represents the pooled effect estimate.

**Figure 6:**
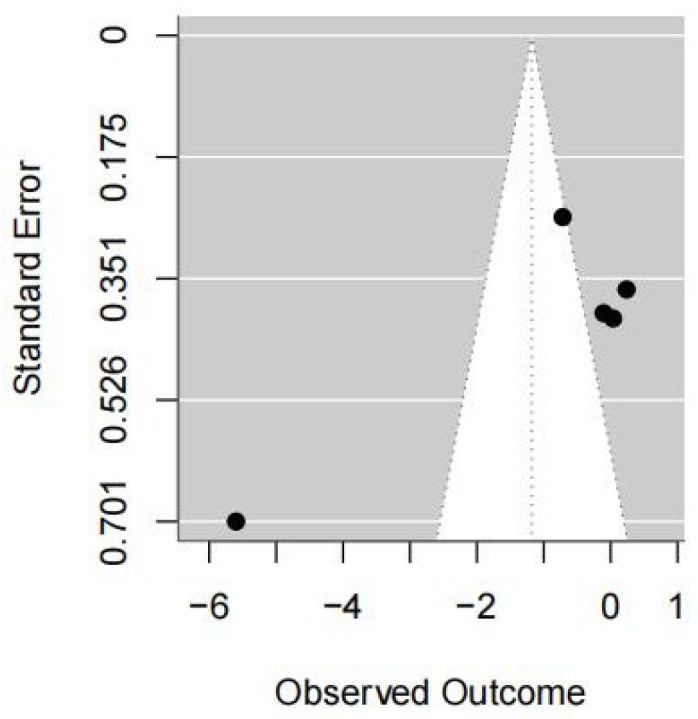
Forest plot of included studies in terms of COP sway path length in the eyes-dclose state. Each dot represents an individual study. The dotted vertical line indicates the pooled effect estimate, and the grey shaded area represents the 95% confidence region of the funnel plot.

Five studies were included in this meta-analysis. Under the random-effects model, the VR intervention was associated with a reduction in COP sway path length under eyes-closed conditions, with a pooled effect size of SMD = −0.54 (95% CI −0.87 to −0.22; I^2^ = 93%; *p* = 0.001) (Figure 5 and Figure 6).

Sensitivity analyses using alternative estimators of between-study variance showed a similar direction of effect; however, confidence intervals crossed the null value and statistical significance was not consistently observed (REML: 95% CI −3.26 to 0.91, *p* = 0.27; DL: 95% CI −2.44 to 0.22, *p* = 0.10) (Table 2).

The leave-one-out analysis indicated that the pooled effect size and its confidence interval remained consistent across all scenarios (ranging approximately from −4.11∼−0.67), and none of the results reached statistical significance (*p* > 0.05). Zhang (2020) [44] contributed substantially to the heterogeneity (I^2^ decreased from 90% to 40% when excluded). This suggests that the high I^2^ was largely driven by this study (Table 2).

Overall, the direction of the pooled effect remained consistent; however, the heterogeneity was influenced by the inclusion of Zhang (2020) [44], indicating sensitivity in this regard.

#### 3.5.3. COP Velocity with Eyes Open

**Figure 7:**
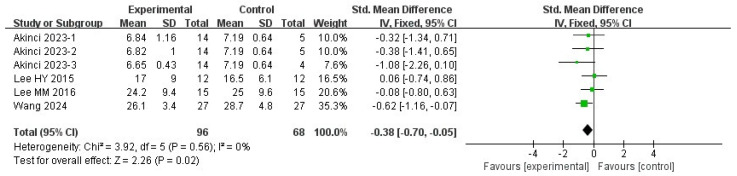
Standardized mean difference for the effectiveness of VR intervention compared with non-VR treatment for COP velocity with open eyes. Green squares indicate individual study effects, with square size proportional to study weight, and horizontal lines represent 95% confidence intervals; the diamond represents the pooled effect estimate.

**Figure 8:**
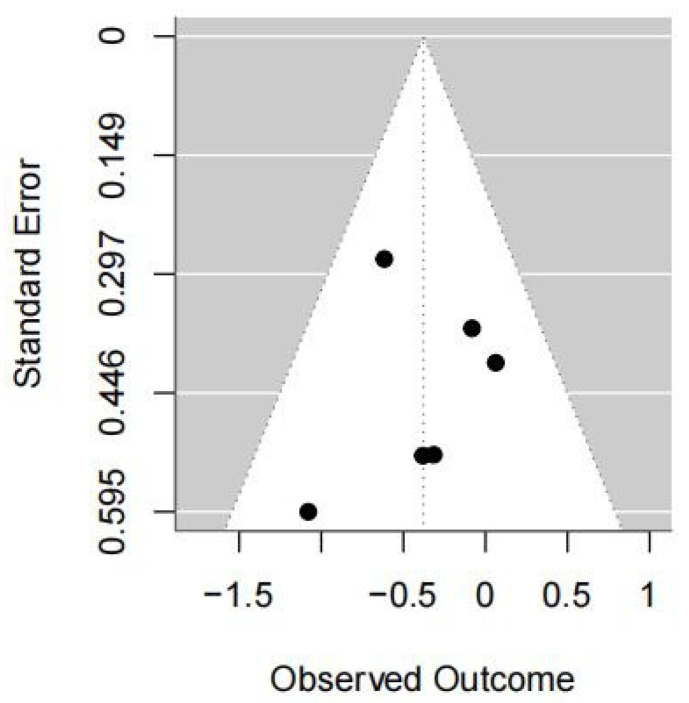
Forest plot of included studies in terms of COP velocity with open-eyes state. Each dot represents an individual study. The dotted vertical line indicates the pooled effect estimate, and the grey shaded area represents the 95% confidence region of the funnel plot.

Four studies were included in this meta-analysis. The VR intervention significantly improved COP velocity under eyes-open conditions in patients with stroke, with a pooled effect size of SMD = −0.38 (95% CI −0.70 to −0.05; I^2^ = 0%; *p* = 0.02) (Figure 7 and Figure 8).

Sensitivity analyses using different estimators of between-study variance yielded identical results, with pooled effect sizes of −0.38 (95% CI −0.70 to −0.05; *p* = 0.02) under both the REML and DL methods, indicating that the findings were not sensitive to the choice of variance estimator (Table 3).

Leave-one-out sensitivity analyses showed that the pooled effect size remained generally stable when individual studies were sequentially excluded. In most scenarios, heterogeneity remained very low (I^2^ = 0%). When Akinci (2023-1) [36] or Akinci (2023-2) [36] was removed, heterogeneity increased slightly (I^2^ = 2.15% and 2.68%, respectively), although these changes did not materially affect the pooled estimates. However, exclusion of Wang (2024) [34] or Akinci (2023-3) [36] resulted in confidence intervals crossing the null value, indicating reduced statistical certainty under these scenarios rather than a reversal of the effect direction (Table 3).

Overall, the results were consistent across analytical approaches, but should be interpreted cautiously due to the limited number of included studies and the sensitivity observed in leave-one-out analyses.

#### 3.5.4. COP Velocity with Eyes Closed

**Figure 9:**
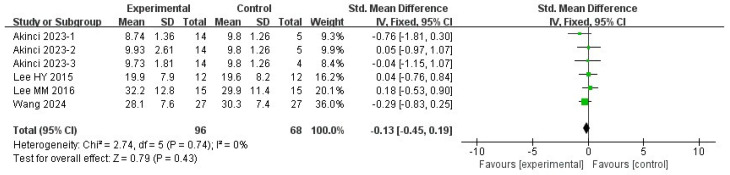
Standardized mean difference for the effectiveness of VR intervention compared with non-VR treatment for COP velocity with closed eyes. Green squares indicate individual study effects, with square size proportional to study weight, and horizontal lines represent 95% confidence intervals; the diamond represents the pooled effect estimate.

**Figure 10:**
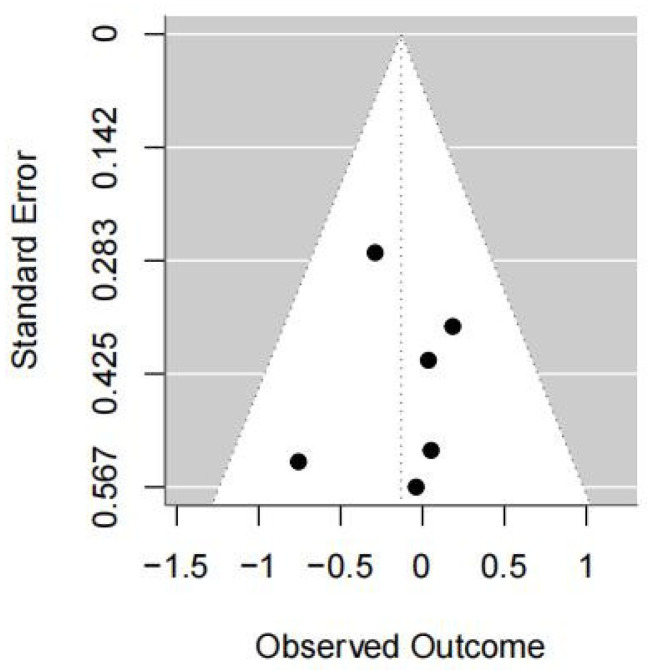
Forest plot of included studies in terms of COP velocity with closed-eyes state. Each dot represents an individual study. The dotted vertical line indicates the pooled effect estimate, and the grey shaded area represents the 95% confidence region of the funnel plot.

Four studies were included in this meta-analysis. Under the random-effects model, the VR intervention was not associated with a statistically significant change in COP velocity under eyes-closed conditions in patients with stroke (SMD = −0.13, 95% CI −0.45 to 0.19; I^2^ = 0%; *p* = 0.43) (Figure 9 and Figure 10).

Sensitivity analyses using different estimators of between-study variance yielded similar results, with pooled effect sizes of −0.13 (95% CI −0.45 to 0.19; *p* = 0.43) under both the REML and DL methods, indicating that the findings were not sensitive to the choice of variance estimator (Table 4).

The leave-one-out sensitivity analysis indicated that the pooled effect size and its confidence interval remained consistent across all scenarios (ranging approximately from −0.57 to −0.40), and none of the results reached statistical significance (*p* > 0.05). Heterogeneity remained 0% in all cases, suggesting that the overall findings were stable and not driven by any single study (Table 4).

Overall, the results indicate that no clear evidence of an effect on COP velocity under eyes-closed conditions was observed in the current analysis. These findings should be interpreted with caution, given the small number of included studies, which may limit the ability to detect true effects or between-study heterogeneity.

### 3.6. Secondary Outcome: Dynamic Balance

#### 3.6.1. Berg Balance Scale Results

**Figure 11:**
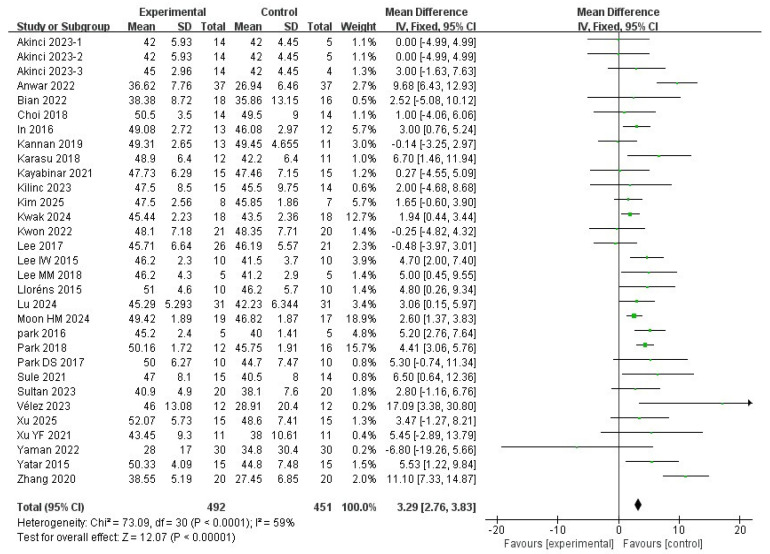
Mean difference for the effectiveness of VR intervention compared with non-VR treatment for Berg Balance Scale. Green squares indicate individual study effects, with square size proportional to study weight, and horizontal lines represent 95% confidence intervals; the diamond represents the pooled effect estimate.

**Figure 12:**
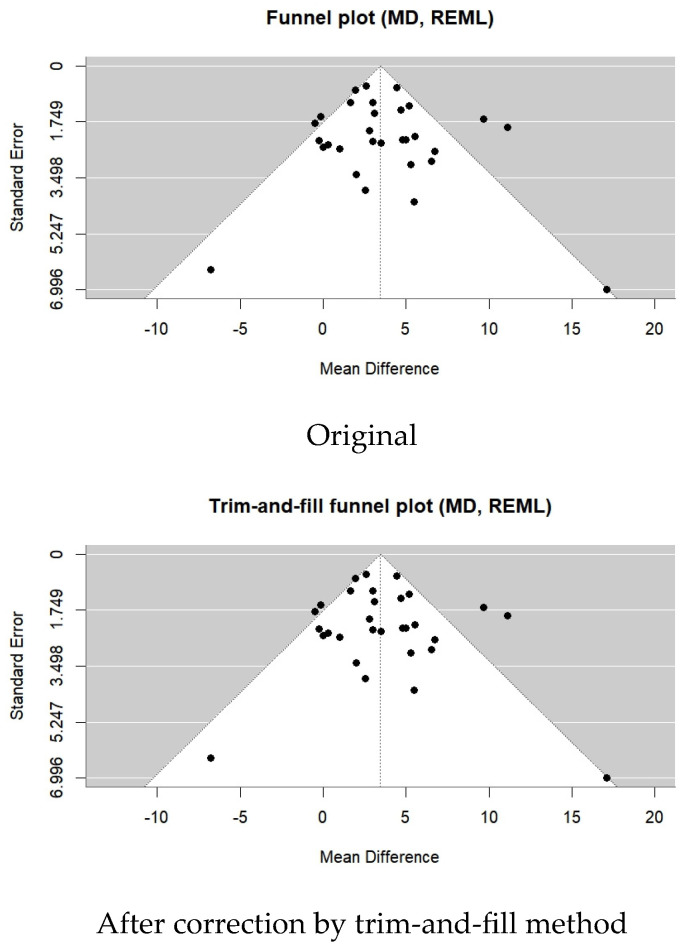
Forest plot of included studies in terms of Berg Balance Scale. Each dot represents an individual study. The dotted vertical line indicates the pooled effect estimate, and the grey shaded area represents the 95% confidence region of the funnel plot.

A total of 29 studies were included in this meta-analysis. The BBS score of patients with stroke significantly improved after the VR intervention (MD = 3.29; 95% CI: 2.76∼3.83; I^2^ = 59%; *p* < 0.00001) (Figure 11 and Figure 12).

Publication bias was assessed using Egger’s regression test based on mean difference (MD). The analysis did not indicate significant small-study effects (z = 0.30; *p* = 0.77; b = 3.14; CI: 0.80∼5.48). No additional studies were imputed using the trim-and-fill method.

#### 3.6.2. Timed Up-And-Go Test Results

**Figure 13:**
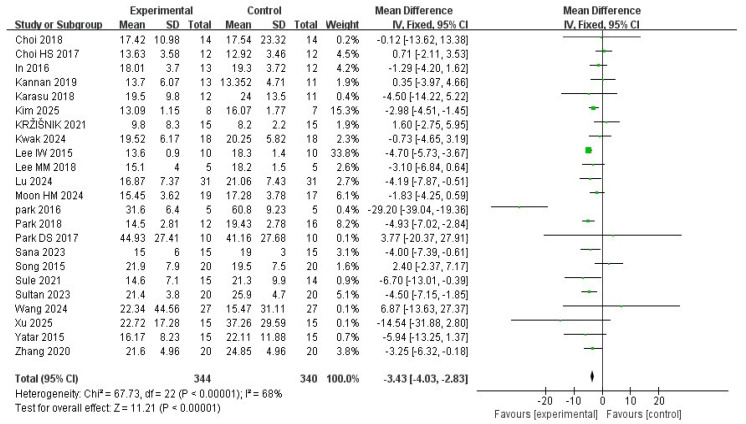
Mean difference for the effectiveness of VR intervention compared with non-VR treatment for the timed up-and-go test. Green squares indicate individual study effects, with square size proportional to study weight, and horizontal lines represent 95% confidence intervals; the diamond represents the pooled effect estimate.

**Figure 14:**
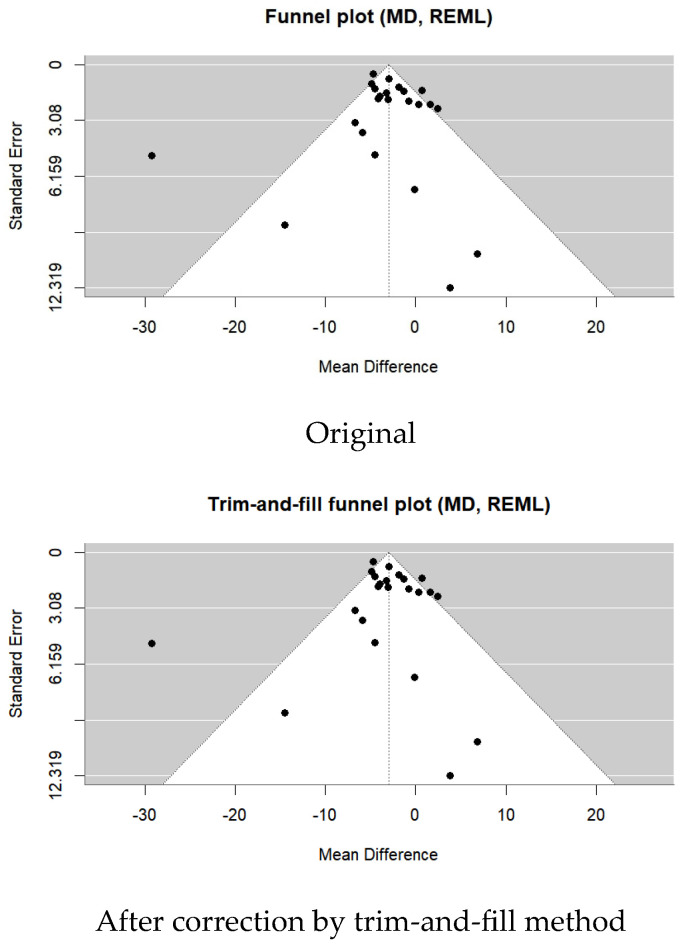
Forest plot of included studies in terms of the timed up-and-go test. Each dot represents an individual study. The dotted vertical line indicates the pooled effect estimate, and the grey shaded area represents the 95% confidence region of the funnel plot.

A total of 23 studies were included in the meta-analysis. The TUG test of stroke patients significantly improved after the VR intervention (MD= −3.43; 95% CI: −4.03∼−2.82; I^2^ = 68%; *p* < 0.00001) (Figure 13 and Figure 14).

Publication bias was assessed using Egger’s regression test based on mean difference (MD). The analysis did not indicate significant small-study effects (z = −1.01; *p* = 0.31; b = −2.04; CI: −4.38∼0.29). No additional studies were imputed using the trim-and-fill method.

### 3.7. Meta-Regression Analysis

Meta-regression analysis was performed on the BBS (31 included studies) and TUG (23 included studies) to account for heterogeneity in the meta-analysis. The intervention time, stroke type, intervention type, VR tool used at the time of intervention, and publication time could not explain the potential heterogeneity in both outcomes (Table 5).

### 3.8. GRADE

The results of the meta-analysis and descriptive analysis were used for GRADE evaluation. The GRADE evaluation of static postural control was downgraded from high to very low, and the GRADE evaluation of dynamic balance was downgraded from high to low. The static postural control results were mainly attributable to the risk of bias, inconsistency, and imprecision in the included studies. The dynamic balance results were mainly attributed to the risk of bias in the included studies and publication bias (Table 6).

## 4. Discussion

### 4.1. Principal Findings

This meta-analysis examined the effects of VR training on balance in patients with stroke from two perspectives. A total of 36 studies from nine different countries involving 1118 participants were included. This study is, to our knowledge, the first meta-analysis to systematically examine static postural control outcomes using center of pressure parameters in stroke rehabilitation. According to the meta-analysis results, patients with stroke in the VR intervention group generally demonstrated better static postural control and dynamic balance outcomes compared with those in the control group. Across the included analyses, the pooled effect estimates were predominantly in favor of the VR intervention.

### 4.2. Effectiveness of VR Interventions on Static Postural Control in Stroke

The effectiveness of static postural control after the VR intervention in stroke patients was considered the primary outcome of this meta-analysis. Initial pooled analyses suggested the effect size was moderate for the two outcomes of eye opening and eye closed movement distance of the COP, SMD = −0.59 (95% CI: −0.89, −0.28; *p* = 0.0002) and SMD = −0.54 (95% CI: −0.87 to −0.22; *p* = 0.001). However, sensitivity analyses indicated that these findings were dependent on the choice of statistical model, with statistical significance not consistently maintained under alternative variance estimators. In the heterogeneity test, the two subgroups showed high heterogeneity (78% and 93%, respectively). However, heterogeneity decreased significantly (41% and 46%, respectively) after removing the Zhang (2020) [44], although the combined effect size was no longer statistically significant.

The effect of the VR intervention was significant only in the eye open state in the center of gravity movement speed, with a moderate effect size (SMD = −0.38, *p* = 0.02) and sensitivity analyses indicated limited robustness. With the eyes closed, there was no difference between the VR intervention group and control group (SMD = −0.13, *p* = 0.43). Sensitivity analyses but influence analyses indicated limited robustness, as individual studies affected the statistical significance of the pooled effect. The heterogeneity was 0% for both subgroups. Although we tried our best to search for and include existing studies, the sample size was relatively small, which may have been the main reason for this result.

Overall, while these findings suggest a potential benefit of VR-based interventions for certain aspects of static postural control, particularly under visually challenging conditions, the evidence remains limited and uncertain. Given the small sample sizes, substantial heterogeneity, and high risk of bias, the certainty of evidence for static postural control outcomes was downgraded to very low. Consequently, these results should be interpreted cautiously and cannot be readily generalized to routine clinical practice. Further large scale, methodologically rigorous trials are required to clarify the role of VR in improving static postural control after stroke.

### 4.3. Effectiveness of VR Interventions on Dynamic Balance in Stroke

The effectiveness of dynamic balance following the VR intervention was considered a secondary outcome in this meta-analysis. Two dynamic balance measures, the BBS and the TUG test, were included. The VR intervention was associated with higher BBS scores (MD = 3.29; *p* < 0.00001) and shorter TUG times (MD = −3.43; *p* < 0.00001) compared with control conditions.

Although statistically significant improvements were observed for both the Berg Balance Scale (BBS) and the Timed Up and Go (TUG) test, Egger’s regression analyses based on mean difference did not indicate significant publication bias for either outcome. Consistently, trim-and-fill analyses did not impute any additional studies and did not materially alter the pooled estimates. Nevertheless, substantial heterogeneity was observed for both outcomes, which, together with methodological limitations and risk of bias across included studies, limits the certainty of these findings. Accordingly, for the dynamic balance outcome (34 RCTs), the certainty of evidence was downgraded from high to moderate.

Overall, statistically significant improvements were observed in BBS and TUG, the clinical meaningfulness of these effects should be interpreted cautiously, as not all pooled effect sizes may reach established minimal clinically important differences (MCIDs), particularly across patients with varying baseline functional levels.

Therefore, to explain the sources of heterogeneity of the two results, this study also conducted five meta-regression analyses, namely the continuous variable intervention time (minutes), dichotomous variable patient stroke type (non-chronic stroke, chronic stroke), type of VR intervention (single VR intervention, VR intervention in combination with other interventions), and type of VR intervention. VR intervention instrument (Nintendo Wii, Nintendo Wii), years of study (<2020, ≥2020). However, none of the five regression analyses accounted for the sources of heterogeneity. Thus, the source of heterogeneity is very complex, and the included studies may have great differences in the setting of intervention methods. Moreover, many studies have vague descriptions of the stroke types of the included participants, which also leads to a lack of fine classification.

### 4.4. Comparison with Previous Studies

VR interventions were associated with better static postural control and dynamic balance outcomes compared with conventional rehabilitation in patients with stroke, which is broadly consistent with findings from previous studies [72,73,74,75,76]. In those studies, improvements in BBS and TUG outcomes were generally observed in the VR groups compared with control conditions. However, most individual studies included fewer than 20 participants per group, and the pooled effect estimates for TUG reported in previous studies did not exceed those observed in the present meta-analysis. Our study included a wider range of studies and yielded a larger effect size. However, earlier meta-analyses have shown that VR interventions do not have better balance effects than conventional interventions [77,78]. These discrepancies may reflect differences in VR technology, intervention design, and clinical implementation across study periods, rather than a uniform improvement attributable solely to technological advancement. The results of three meta-regression analyses in one study showed that the effects of the VR intervention were not related to the VR intervention content, control group type, or VR immersion [72]. This finding was similar to the results of the five meta-regression subgroups in the present study, in which the effect of the VR intervention was not related to the intervention time, stroke type, VR intervention type, VR intervention tool, or publication year. These results are consistent with those of several previous studies showing that the current study could not account for sources of heterogeneity by intervention duration, stroke severity, VR immersion, or device type [18,79]. However, fully immersive and task-specific devices are often associated with better outcomes [80]. Stroke duration has an independent effect on functional outcomes, such as the Fugl–Meyer test [81]. This indicates the need to develop more precise rehabilitation goals and match devices more closely to rehabilitation strategies in stroke treatment research based on VR interventions. In particular, intervention tools, as more of the included studies used low-immersion tools, and a single category (Nintendo Wii Fit) accounted for a relatively high proportion. In the future, with the development of VR technology, more detailed tool classification and effect verification can be performed after the equipment that matches the target is entered into the trial.

This study is, to our knowledge, the first meta-analysis to systematically examine static postural control outcomes using center of pressure parameters in stroke rehabilitation. Some studies have highlighted the assessment of trunk control in an unstable sitting posture on a seesaw is a reliable test for assessing the trunk control ability of individuals after a stroke. The COP length and mean velocity were considered the best parameters [82]. This also indicated a significant correlation between postural control and moving speed of the center of gravity. However, few precise and specialized measures of postural control are available in current stroke-controlled trials using VR interventions. This also leads to an imprecise and unstandardized description of the measurement process when making measurements. A review of the central variables of stress that quantify standing balance in older adults also noted a lack of homogeneity and standardization across studies [83]. Earlier reviews of the effects of other types of interventions, such as exercise therapy, on balance ability in patients with stroke were also rarely included as important results, but the included studies were very few [84]. In several recent reviews on improving balance in patients with stroke, COP is being considered as a more reliable measure [85].

Beyond therapy delivery, virtual rehabilitation is increasingly being integrated with digital assessment of neurological and functional parameters, reflecting a broader trend toward the digitalization of elements of the neurological examination. This article has highlighted how sensor derived and digitally captured metrics may enable more objective, scalable and longitudinal evaluation of neurological function [86]. In this article, COP measures of postural control synthesized in the present review may be viewed not only as outcome measures but also as potential digital biomarkers for balance impairment and recovery in stroke rehabilitation. Future VR rehabilitation systems may therefore combine task specific training with integrated digital assessment, facilitating personalized intervention and continuous monitoring of recovery trajectories.

### 4.5. Limitations

Our study had some limitations. First, although we diligently searched, the sample size of RCTs included in the static postural control was relatively small, and even the area data of the COP were insufficient to be included in the final analysis, which may limit the generality and comprehensiveness of the results. Second, Despite the use of meta-regression, the sources of heterogeneity could not be fully explained. This is likely attributable to substantial clinical heterogeneity, including differences in stroke severity, baseline balance function, cognitive status, and sensory deficits, which were inconsistently reported across the included trials. Such variability may have limited the explanatory power of meta-regression and should be considered when interpreting the pooled estimates. Finally, owing to economic reasons for VR intervention at the application level, there are few related studies in backward or developing countries; therefore, the promotion scope of the meta-analysis results is limited. In the future, attention should be paid to the application and research of VR interventions in the balance treatment of stroke patients in a wide range of countries and regions.

### 4.6. Future Recommendations

Future studies should first clarify the name of the instrument and specific software model used for the VR intervention. In addition, the process description of the VR intervention was more detailed in the development of the research methods, such as adding pictures or video descriptions of the intervention process. The timing and frequency of interventions should be strictly defined and implemented. The affected side (left/right), stroke type (cerebral hemorrhage/cerebral infarction), cognitive level, and balance level (BBS) of the enrolled patients were recorded. In addition, the implementation of placebo VR training for participants and practitioners was blinded. In the subsequent meta-analysis, a more detailed grouping meta-analysis or meta-regression analysis was conducted to reduce heterogeneity and make the research results more accurate for application and promotion in clinical medicine. In addition to a rigorous method design, the design of intervention forms must be flexible and diverse. VR can be used as a carrier for other mature and effective intervention methods to conduct more interesting and acceptable interventions. For example, VR feedback therapy combined with mirror therapy or virtual catch training combined with center-of-gravity transfer training can be used as a development direction for future VR intervention personalization. Second, in the measurement process, a measurement blinding method must be implemented. The description and implementation of measurement methods reduce the need for subjective factors in the evaluation process of outcome measurements. In addition, it is necessary to standardize the measurement process, such as clarifying the name and type of COP measurement tool and specifying the measurement action, standard measurement time, measurement times, and value range. Finally, subsequent effective follow-up should be included in the trial. For example, the fall rate follow-up between the VR intervention group and the general control group at the end of the intervention or the balance level follow-up at one–six months after the intervention. In the future, this can be used as a basis for judging the stability of the effects of VR interventions in the time dimension. It is important to note that the term “virtual reality” encompasses a wide range of interventions that differ substantially in immersion level, task specificity, feedback modality, and therapeutic intent. Some interventions primarily target task specific balance training, whereas others are more game-based or low-immersion, which may engage different motor and cognitive mechanisms. Pooling these heterogeneous interventions under a single VR category may therefore oversimplify their effects and contribute to between-study heterogeneity. This variability should be considered when interpreting the pooled results and highlights the need for more standardized reporting and classification of VR interventions in future trials.

Stroke has long been considered a disease with complex causes, diverse rehabilitation goals, and lengthy processes [87,88]. Particularly for balance rehabilitation in stroke patients, the acute stage is generally treated conservatively, and the subacute and chronic stages are the main periods of balance rehabilitation [89]. As an interesting and reproducible intervention, VR can be promoted and used during rehabilitation. As a rapidly developing treatment, it is commonly used as an adjunctive therapy in the rehabilitation of stroke patients [20,72]. Many patients with stroke have the potential to use VR interventions for rehabilitation [90]. However, with ongoing technological standardization and development, VR interventions may have the potential to play a more prominent role in balance rehabilitation for stroke patients in the subacute and chronic phases. Such approaches could contribute to more standardized training environments and support the delivery of consistent rehabilitation tasks across settings, while potentially reducing physical burden during therapy sessions. Nevertheless, VR is more appropriately viewed at present as a complementary tool rather than a replacement for conventional rehabilitation. Future research should focus on developing more targeted and adaptable VR treatment protocols, enabling individualized intervention strategies tailored to patients with different clinical stages, functional levels, and rehabilitation goals.

## 5. Conclusions

VR-based interventions may be associated with improvements in static postural control and, to a lesser extent, dynamic balance in patients with stroke. However, these findings particularly those related to dynamic balance should be interpreted with caution due to substantial heterogeneity, potential publication bias, and the overall risk of bias among the included trials, which limit the certainty and generalizability of the evidence. Current evidence suggests that VR may serve as a potentially beneficial adjunct to conventional rehabilitation rather than a standalone therapy. Further high-quality, well-controlled trials with standardized intervention protocols and rigorous methodological designs are required to clarify the magnitude, consistency, and clinical applicability of VR-based interventions across diverse stroke populations.

## Figures and Tables

**Table 1:** Model sensitivity results and leave one-out results in terms of COP sway path length in the eye-open state.

Omitted Study	Studies Number	Effect Size (95% CI)	I^2^	Tau^2^	Q	*p*
**verall-REMLO**	5	−1.25∼0.14	79.90%	0.50	18.32	0.12
**Overall-DL**	5	−1.23∼0.11	78.16%	0.45	18.32	0.10
**In 2016 [57]**	4	−1.50∼0.27	85.06%	0.69	17.64	0.17
**Lee HY 2015 [62]**	4	−1.51∼0.11	82.38%	0.56	15.20	0.09
**Lee MM 2016 [53]**	4	−1.52∼0.09	80.96%	0.54	14.01	0.08
**Lu 2024 [33]**	4	−1.42∼0.41	82.73%	0.72	17.82	0.28
**Zhang 2020 [44]**	4	−0.73∼0.17	40.19%	0.08	4.79	0.22

**Table 2:** Model sensitivity results and leave-one-out results in terms of COP sway path length in the eyes-closed state.

Omitted Study	Studies Number	Effect Size (95% CI)	I^2^	Tau^2^	Q	*p*
**Overall-REML**	5	−3.26∼0.91	97.33%	5.45	60.15	0.27
**Overall-DL**	5	−2.44∼0.22	93.35%	2.10	60.15	0.10
**In 2016 [57]**	4	−4.10∼1.17	97.89%	7.01	58.56	0.28
**Lee HY2015 [62]**	4	−4.10∼1.11	97.87%	6.87	57.55	0.26
**Lee MM2016 [53]**	4	−4.11∼1.02	97.64%	6.64	54.10	0.24
**Lu 2024 [33]**	4	−4.03∼1.40	94.47%	7.44	59.58	0.34
**Zhang2020 [44]**	4	−0.67∼0.28	46.85%	0.11	5.58	0.42

**Table 3:** Model sensitivity results and leave-one-out results in terms of COP velocity in the eyes-open state.

Omitted Study	Studies Number	Effect Size (95% CI)	I^2^	Tau^2^	Q	*p*
**Overall-REML**	6	−0.70∼−0.05	0%	0	3.96	0.02
**Overall-DL**	6	−0.70∼−0.05	0%	0	3.96	0.02
**Akinci 2023-1 [36]**	5	−0.73∼−0.04	2.15%	0	3.94	0.03
**Akinci 2023-2 [36]**	5	−0.72∼−0.03	2.68%	0.01	3.96	0.04
**Akinci 2023-3 [36]**	5	−0.66∼0.02	0%	0	2.45	0.07
**Lee HY 2015 [62]**	5	−0.82∼−0.11	0%	0	2.57	0.01
**Lee MM 2016 [53]**	5	−0.82∼−0.09	0%	0	3.14	0.02
**Wang 2024 [34]**	5	−0.65∼0.16	0%	0	2.82	0.23

**Table 4:** Model sensitivity results and leave-one-out results in terms of COP velocity in the eyes-close state.

Omitted Study	Number of Studies	Effect Size (95% CI)	I^2^	Tau^2^	Q	*p*
**Overall-REML**	6	−0.45∼0.19	0%	0	2.76	0.43
**Overall-DL**	6	−0.45∼0.19	0%	0	2.76	0.43
**Akinci 2023-1 [36]**	5	−0.40∼0.27	0%	0	1.25	0.71
**Akinci 2023-2 [36]**	5	−0.49∼0.19	0%	0	2.63	0.38
**Akinci 2023-3 [36]**	5	−0.47∼0.20	0%	0	2.74	0.42
**Lee HY 2015 [62]**	5	−0.51∼0.19	0%	0	2.57	0.37
**Lee MM 2016 [53]**	5	−0.57∼0.15	0%	0	1.84	0.25
**Wang 2024 [34]**	5	−0.44∼0.36	0%	0	2.24	0.84

**Table 5:** Meta-regression covariates of VR therapy on static postural control and dynamic balance in patients with stroke.

Covariates	Estimate	SE	Z Value	*p* Value (95% CI)
**BBS**				
Intervention time (min)	0.000	0.000	0.083	0.934 (−0.0002, 0.0002)
**Stroke stage (chronic stroke or no chronic stroke)**	−0.118	0.238	−0.496	0.620 (−0.5843, 0.3481)
**Intervention (VR alone or no VR intervention alone)**	0.035	0.243	0.145	0.884 (−0.4410, 0.5117)
**Intervention VR tool (Nintendo Wii Fit or no Nintendo Wii Fit)**	0.108	0.259	0.416	0.678 (−0.3991, 0.6142)
**Year of publication (<2020 or ≥2020)**	−0.298	0.242	−1.233	0.218 (−0.7725, 0.1759)
**TUG**				
Intervention time (min)	0.0001	0.0002	0.773	0.439 (−0.0002, 0.0005)
**Stroke stage (chronic stroke or no chronic stroke)**	−0.336	0.327	−1.027	0.304 (−0.9765, 0.3050)
**Intervention (VR Alone—No VR intervention alone)**	0.189	0.34	0.556	0.578 (−0.4766, 0.8542)
**Intervention VR tool (Nintendo Wii Fit—No Nintendo Wii Fit)**	−0.298	0.345	−0.863	0.388 (−0.9742, 0.3784)
**Year of publication (<2020 or ≥2020)**	−0.09	0.327	−0.276	0.783 (−0.7314, 0.5508)

**Table 6:** Level of evidence for VR training of static postural control and dynamic balance in stroke rehabilitation patients.

Outcomes and Number of Studies	Risk of Bias	Inconsistency	Indirectness	Imprecision	Publication Bias	Quality of the Evidence (GRADE)	Comments
**Static postural control (7 RCTs)**						Very low	The VR intervention group had a moderate effect size and showed statistical effects in static posture control. However, owing to the small number of included studies and the difficulty in blinding the experimental process because of the implementation of VR intervention, the overall evidence was very low
**Center of pressure sway path length with open eyes (5 RCTs)**	Approximately 80% of the studies had a high risk of bias, which was reduced by two levels	I^2^ = 78%, 40% of the effect size directions are inconsistent, one level lower	No degradation	The sample size was small (*n* = 181), and the CI (−0.89, −0.29) did not have a zero-crossing effect, resulting in a one -level reduction	The number of included studies is <10 and cannot be evaluated. It will not be downgraded.	Very low	
**Center of pressure sway path length with close eyes (5 RCTs)**	Approximately 80% of the studies had high risk of bias, which was reduced by two levels	I^2^ = 93%, 16% of the effect size directions are inconsistent, and there is no degradation	No degradation	The sample size was small (*n* = 181), and the CI (−0.87, −0.22) did not have a zero-crossing effect, resulting in a one-level reduction	The number of included studies is <10 and cannot be evaluated. It will not be downgraded.	Very low	
**Center of pressure velocity with open eyes (4 RCTs)**	Approximately 75% of the studies had high risk of bias, which was reduced by two levels	I^2^ = 0%, the effect size direction is 40% inconsistent, reducing by one level	No degradation	The sample size was small (*n* = 164), and the CI (−0.70, −0.05) did not cross the 0 effect, resulting in a one-level reduction	The number of included studies is <10 and cannot be evaluated. It will not be downgraded.	Very low	
**Center of pressure velocity with closed eyes (4 RCTs)**	Approximately 75% of the studies had high risk of bias, which was reduced by two levels	I^2^ = 0%, 50% of the effect size directions are inconsistent, one level lower	No degradation	The sample size was small (*n* = 164), and the CI (−0.45, 0.19) crossed the 0 effect, reducing by two levels	The number of included studies is <10 and cannot be evaluated. It will not be downgraded.	Very low	
**Dynamic balance (34 RCTs)**						moderate	The results all showed large effect sizes and significant statistical significance. However, owing to fact that most of the included experiments were difficult to implement blinding during the experimental process, the level of evidence was moderate.
**BBS (31 RCTs)**	Approximately 40% of the studies had a high risk of bias, which was reduced by one level	I^2^ = 59%, 13% of the effect size directions are inconsistent, and removing any one of the leave one out has no impact on the effect size and does not downgrade	No degradation	The sample size is sufficient (*n* = 943), and the CI (2.76, 3.83) does not have a zero-crossing effect and does not downgrade	No Publication bias	moderate	
**TUG (23 RCTs)**	Approximately 48% of the studies had a high risk of bias, which was reduced by one level	I^2^ = 68%, 26% of the effect size directions are inconsistent, and removing one or two one by one has no impact on the effect size and does not downgrade	No degradation	The sample size is sufficient (*n* = 694), and the CI (−4.03, 2.82) does not have a zero-crossing effect and does not downgrade	No Publication bias	moderate	

## Data Availability

No new data were created or analyzed in this study.

## Supplementary Materials

The following supporting information can be downloaded at: https://www.mdpi.com/article/10.3390/medicina62010090/s1.

## Abbreviations

BBS: Berg Balance Scale; COP: center of pressure; DL: DerSimonian–Laird; EO: eyes open; EC: eyes closed; GRADE: Grading of Recommendations Assessment, Development and Evaluation; MD: mean difference; REML: restricted maximum likelihood; SMD: standardized mean difference; TUG: Timed Up and Go Test; VR: virtual reality.
